# Inflammatory Bowel Diseases: It's Time for the Adenosine System

**DOI:** 10.3389/fimmu.2020.01310

**Published:** 2020-07-29

**Authors:** Luca Antonioli, Matteo Fornai, Carolina Pellegrini, Lorenzo Bertani, Zoltan H. Nemeth, Corrado Blandizzi

**Affiliations:** ^1^Department of Clinical and Experimental Medicine, University of Pisa, Pisa, Italy; ^2^Department of Pharmacy, University of Pisa, Pisa, Italy; ^3^Department of Translational Research and New Technologies in Medicine and Surgery, University of Pisa, Pisa, Italy; ^4^Department of Surgery, Morristown Medical Center, Morristown, NJ, United States; ^5^Department of Anesthesiology, Columbia University Medical Center, New York, NY, United States

**Keywords:** adenosine, adenosine receptor, immune system, intestinal motility disorder, inflammatory bowel diseases, novel drugs and targets

## Introduction

### The Adenosine System: Enzymes, Transporters, and Receptors

Over the years, a number of evidences have pointed out the relevant contribution of the adenosine system in the regulation of different physiological functions, highlighting a deep involvement of this nucleoside in shaping the digestive functions (1). Of note, such modulatory effects are tightly related to the levels reached by this nucleoside in the biophase of its own receptors (2). In this regard, it has been well reported that the adenosine levels vary considerably based on the health status of the tissues (3).

Under physiological conditions, low levels of adenosine are detected in the extracellular milieu, which stems mainly from the intracellular activity of S-adenosylhomocysteine hydrolase, which converts the S-adenosylhomocysteine into adenosine (1). Once synthesized, adenosine is extruded from the cells via nucleoside transporters, classified into: (a) equilibrative nucleoside transporters (ENTs), bidirectional transporters, acting on the intra- and extracellular levels reached by the nucleoside; (b) the concentrative nucleoside transporters (CNTs), promoting the intracellular influx of adenosine against its concentration gradient. Once re-uptaken intracellularly, adenosine is quickly phosphorylated into adenosine monophosphate (AMP) by adenosine kinase or deaminated into inosine via the catabolic enzyme adenosine deaminase (1). Under pathological conditions, the extracellular levels of adenosine increased markedly, mainly via the up-regulation of the ecto-nucleoside triphosphate diphosphohydrolase 1 (CD39)-5′-nucleotidase (CD73) enzyme axis, which quickly convert the extracellular adenosine 5'-triphosphate (ATP) into adenosine (4).

The extracellular levels of adenosine are tightly controlled by adenosine deaminase which converts this nucleoside into inosine, and then to the end product uric acid via xanthine oxidase (5). In parallel, adenosine kinase also takes part to finely tune the extracellular adenosine concentration, phosphorylating it once recovered inside the cell (1).

The physiological and pathophysiological activity of adenosine are mediated by the engagement of four specific G-protein-coupled receptors named A_1_, A_2A_, A_2B_, and A_3_ (6). The A_1_ and A_3_ receptors, once stimulated, induce an intracellular release of calcium via interaction with G_i_, G_q_, and G_o_ proteins (7). The A_2A_ and A_2B_ receptors, related with G_s_ or G_olf_, activate adenylyl cyclase (7). Of note, A_2B_ receptors can also elicit the activation of the phospholipase C via G_q_ protein (7).

### Adenosine System in IBD Pathophysiology

IBDs are chronic relapsing disorders affecting the digestive tract, clinically classified as Crohn's disease or ulcerative colitis based on symptoms, disease location, and histopathological features (8).

A common denominator observed in IBD patients is deregulated intestinal mucosa functions as well as an exuberant activity of immune cell populations (9, 10). Indeed, in IBD patients the barrier function appears critically compromised, thus leading to an increased permeability to noxious intraluminal stimuli. In particular, the bacteria, overwhelming the intestinal barrier, infiltrate the *lamina propria* triggering the mucosal immune system activity and spurring the inflammatory process (11). In parallel, the IBD patients display both a T cell dysfunction as well as an antigen-presenting cell alteration (9, 10).

Clinically, it has been reported that Crohn's disease can affect any part of the digestive tract (12). In particular, this disorder is characterized by “patches,” affecting some areas of the gut, leaving other sections completely unaltered (12). Histologically, Crohn's disease displays a transmural inflammation of the bowel wall (12). By contrast, ulcerative colitis is limited to the colon and the rectum, with an inflammation occurring only in the innermost layer of the lining of the intestine (12).

Immunologically, Crohn's disease is characterized by a T_H1_/T_H17_ paradigm, leading to a marked release of IL-1β, IL-6, IL-12, IL-17, IL-21, IL-22, IL-23, IL-26, TNF, and IFN-γ (13). The ulcerative colitis patients showed a T_H2_/T_H9_ paradigm, which determines a massive production of IL-4, IL-5, IL-9, IL-13, and IL-25 (13).

Over the years, the increasing availability of different preclinical models of IBDs, allowed a better understanding of the pathophysiological mechanisms underlying these diseases (14). At present, more than 60 animal models have been established to study IBD, distinguished in chemically induced, congenial mutant, cell-transfer, and genetic models (14). Among the chemical-induced colitis models, the dinitro- or trinitrobenzene sulfonic acid (DNBS or TNBS, respectively), oxazolone and dextran sulfate sodium (DSS)-induced colitis are widely employed (15). DNBS- or TNBS-administration elicited a T_H1/_TH_17_ immune response, closely mimicking the Crohn's disease features (15). The DSS-induced colitis, despite being widely employed, is a spurious model, displaying a T_H1_/T_H2_ cytokine pattern (15). By contrast, ulcerative colitis in humans is well mimicked by the oxazolone-induced colitis, which typically exhibits a T_H2_ immune response (15).

Among the genetically engineered murine models of colitis, the IL-10-knockout mice spontaneously develop a transmural pancolitis and cecal inflammation, similar to human Crohn's disease (14). In addition, the adoptive transfer models, induced by the selective transfer of immune cell types, usually CD4^+^ T cells, in immunodeficient animals, provided relevant information about the role of T cells in shaping the mucosal immunity (14).

In parallel, the availability of a number of cell culture systems allowed to *in vitro* dissect the relevance of various cell populations in IBD onset and development (16). However, the cell culture models display several points of criticisms. Indeed, most of the cell lines employed are often immortalized neoplastic cell lines (16). In particular, the colonic cell lines Caco-2, HT29 and T84, despite displaying morphological and functional features of differentiated intestinal epithelial cells, they are characterized by neoplastic features in terms of phenotype and metabolism, thus not adequately representing the physiological or the inflammatory condition (16). In this regard, the primary human intestinal epithelial cells obtained from healthy subjects or IBD patients should be the most representative model, but unfortunately their employment is complicated by the extreme phenotype variability and by the reduced viability once in culture (16).

Over the years, several evidences highlighted a critical role of adenosine in the maintenance of intestinal homeostasis, and in orchestrating the interplay between the intestinal epithelial cells, the neuromuscular compartment and the enteric immune system (1). In particular, adenosine and its receptors demonstrated a profound reorganization in the inflammatory contexts, taking a significant part in shaping the immune responses (17). On these premises, several studies investigated the therapeutic potential of ligands acting on the adenosine system in the management of intestinal inflammation (17). However, a critical evaluation of the available pre-clinical studies about the efficacy of drugs acting on the adenosine system in managing the chronic inflammatory bowel diseases, is complicated by the heterogeneity of the *in vivo* and *in vitro* models employed, which could lead, in some cases, to conflicting results.

### Role of Adenosine System in Intestinal Inflammation

Crohn's patients with active disease displayed an increased A_2A_ receptor mRNA expression in colonic mucosa, while no changes were observed in patients with ulcerative colitis (18). Conversely, others reported a decreased mRNA and protein expression of A_2A_ receptor in sigmoid colonic mucosa from active ulcerative colitis patients (19, 20). In addition, the authors observed that A_2A_ receptor expression was oppositely related with miR-16 expression (19). In particular, miR-16, targeting the 3′-UTR of A_2A_ receptor mRNA, has been found to inhibit A_2A_ receptor transcription (19). Zhang et al. (20) observed a correlation between the increase in miR-15 and a decreased expression of A_2A_ receptor mRNA in colonic tissues from ulcerative colitis patients. The authors demonstrated in HT-29 cell lines that miR-15 downregulated A_2A_ receptor mRNA expression, which, in turn, decreased the activation of pro-inflammatory NF-κB signaling (20).

Several studies showed that in the presence of bowel inflammation, A_2A_ receptors critically regulate T cell functions. Naganuma et al. (21) reported that co-transfer of CD45RB^low^ or CD25^+^ Th cells lacking A_2A_ receptors to immunodeficient mice transferred of pathogenic CD45RB^high^ Th cells failed to prevent disease. Conversely, co-transfer of *wild-type* CD45RB^low^ or CD25^+^ Th cells prevented the onset of the disease, revealing a critical involvement of A_2A_ receptor in the onset of experimental colitis.

The pharmacological activation of A_2A_ receptors, via inosine administration, has exerted beneficial effects in animals with colitis induced by TNBS, indicating the A_2A_ receptor activation as an intriguing pharmacological strategy for management of gut inflammation (22). Likewise, oral administration of PSB-0777, a scarcely absorbed A_2A_ receptor agonist, alleviated bowel inflammation in oxazolone-induced colitis rats (23). However, treatment with CGS21680, a recognized selective A_2A_ receptor agonist failed in ameliorating a murine model of DSS-induced colitis (24).

The discrepancy in term of efficacy about the pharmacological A_2A_ receptor stimulation in the murine models of colitis, could be ascribable to the difference in the pathophysiological mechanisms underlying such experimental models. Indeed, it has been widely recognized that the T cells play a relevant role in the onset and development of TNBS or oxazolone colitis (25, 26), but not in DSS colitis (27). Of note, the main immunomodulatory action of A_2A_ receptors is mainly targeted to T cell population and only marginally on other immune cell populations (28). In line with this evidence, a number of data showed a lack of efficacy of CGS 21680 in stemming the phlogistic process, such as the DSS colitis, mainly driven by macrophages (29, 30).

Besides A_2A_ receptors, enteric immune and non-immune cells, with particular regard for intestinal epithelial cells, express A_2B_ receptors (31, 32). Indeed, both patients and mice with colitis displayed an increased A_2B_ receptor expression in the intestinal epithelial cells (33). In this context, A_2B_ receptors hold a key role in the maintenance of gut epithelial barrier integrity and functions, through the regulation of secretory activity, permeability and interaction with bacteria, pivotal factors implicated in IBD (33). Of note, the endothelial cells and the macrophages also showed the presence of A_2B_ receptors (34). Previous studies showed that the pharmacological block or gene deletion of A_2B_ receptor ameliorated the colitis in mice (35, 36). Conversely, Frick et al. reported that both the genetic or pharmacological ablation of A_2B_ receptors augmented the course of colitis, thus suggesting a protective role for A_2B_ receptors (37). In addition, they demonstrated that mice with A_2B_ receptor gene deletion in intestinal epithelial cell were less susceptible to the development of bowel inflammation, thus confirming a pivotal role of A_2B_ in the protection against colitis, suppression of inflammation as well as in preserving intestinal barrier integrity (38). These conflicting data regarding the role of A_2B_ receptors in bowel inflammation could result from different experimental designs, environmental variability and differences in knockout murine strains, including variation in bacterial flora composition.

Of interest, a role of A_3_ adenosine receptors in the pathophysiological mechanisms of IBDs has also been described. Indeed, both patients with ulcerative colitis and in animals with experimental colitis displayed a decrease in A_3_ receptor expression in colonic tissues (18, 39, 40). However, others reported an increased level of A_3_ receptors in peripheral blood mononuclear cells of Crohn's patients (41). In a recent study, Ren et al. (42) showed that patients with ulcerative colitis were characterized by a decreased A_3_ receptor expression along with an increase in TNF and IL-1β concentrations as well as NF-κB p65 expression in colonic mucosa. The pharmacological stimulation of A_3_ receptors via 2-Cl-IB-MECA reduced the TNF and IL-1β levels and counteracted the NF-κB p65 activation in colonic tissues from UC patients, thus suggesting a role of A_3_ in the pathogenesis of bowel inflammation (42). Mabley et al. (43) demonstrated that IB-MECA treatment to DSS mice, TNBS rats as well as IL-10^−/−^ animals exerted beneficial effects on bowel inflammation, ameliorating the clinical symptoms and histological signs of inflammation and suppressed inflammation (43, 44). Conversely, gene deletion of A_3_ receptor in mice was associated with a lower susceptibility to the development of colitis induced by DSS (40).

Such conflicting findings could be ascribed to different experimental conditions, including differences in gut microbiota composition, regarded as an important factor in the development of colitis (45). In addition, it is worth noting that the ablation of A_3_ adenosine receptors determines an upregulation of other adenosine receptors, such as A_2A_, which, in turn, exert protective effects in bowel inflammation (21).

Of interest, besides the adenosine receptors, the CD39/CD73 axis, involved in the adenosine synthesis, is emerging as a novel pharmacological target in IBD (4).

Gibson et al. observed a decreased expression of CD39 on T_regs_ from IBD patients when compared with healthy subjects (46). In addition, the authors reported that treatment with the anti-TNF infliximab determined an increase in CD39 expression on T_regs_ (46). Bai et al. observed a decreased expression of CD39 in a Th17 subpopulation with suppressor activity of patients with IBDs (47). Others reported an increase in CD39^+^CD8^+^ T cells in peripheral blood as well as in the *lamina propria* of Crohn's disease patients (48). Both CD39^+^ Th17 and CD39^+^CD8^+^ T cells have been found to exert immunosuppressive effects through the production of adenosine (48). Confirming the immunosuppressive role of CD39 in IBDs it has been observed that a single nucleotide polymorphism determining low levels of CD39 expression was related with a higher susceptibility to the development of Crohn's disease in a case-control cohort including 1,748 IBD patients and 2,936 controls (49). Taken together, these findings suggest a protective role of CD39 in patients with IBDs.

To better understand the role of CD39 in the pathogenesis of bowel inflammation has been well characterized by means of animal models of colitis. Friedman et al. showed that CD39^−/−^ mice displayed an enhanced inclination to DSS-induced colitis. Such an effect was rescued by the administration of exogenous ATPase apyrase (49). Conversely, others observed that TNBS mice with CD39 gene deletion were characterized by a lower severity of colitis as compared with wild type TNBS animals (50). In addition, they observed that the severity of oxazolone-induced colitis was comparable in CD39 KO mice as well as in wild-type animals (50). The explanation for these heterogenous results could be ascribed to the different experimental models of colitis employed. Indeed, the TNBS model shows clinicopathological features reminiscent to Crohn's disease while oxazolone-induced colitis resembles ulcerative colitis (26). However, further investigations in mice with cell-specific and temporal targeting of CD39 are needed to clarify the involvement of CD39 in bowel inflammation.

A critical role for CD73 in maintaining intestinal homeostasis has also been described (51–54). Doherty et al. displayed that patients with IBD were characterized by an increased numbers of circulating and colonic CD73^+^CD4^+^ T cells during the active phase of inflammation and such an increase was counteracted following anti-TNF treatment (55). In addition, patients with active IBD displayed an increase in CD73 on Th_17_ cells (55).

In order to better clarify the role of CD73 in the onset of bowel inflammation, several studies have been carried out in pre-clinical models of colitis. One of these works demonstrated an increased expression of CD73 in colonic mucosa of mice treated with TNBS (51). In addition, the induction of colitis in CD73^−/−^ mice was associated with worsening clinical course and inflammation. On the same line, the pharmacological blockade of CD73 with the selective inhibitor α,β-methylene ADP increased the severity of colitis in *wild type* TNBS mice (51). In addition, Bynoe et al. demonstrated that the protective effects of CD73 in bowel inflammation resulted from the induction of IFN-αA, whose administration reversed the deleterious CD73 phenotype (56). However, the activation of CD73 on T_regs_ was not dispensable for its protective effects in bowel inflammation. Moreover, the co-transfer of *wild-type* T_regs_ to Rag^−/−^ mice exerted beneficial effects on bowel inflammation comparable to co-transfer of CD73 deficient T_regs_ (56). Based on these data, it is evident that the relevance of CD73 in the pathophysiology of the intestinal inflammation. In particular, by means of CD73 knockout mice, it has been demonstrated that a reduced expression of this enzyme in effector immune cells contribute to the IBD pathogenesis. Moreover, the critical role of CD73 in the maintenance of the colonic epithelium integrity has been also observed, as corroborated by the marked degree of colonic inflammation and tissue damage in CD73 knockout mice.

Growing evidence highlights an involvement of ADA in the IBD pathophysiology (5). Maor et al. showed that Crohn's patients during the active phase of the disease displayed higher circulating ADA and ADA2 levels in comparison with patients in remission as well as in healthy subjects (57). In addition, the increased circulating ADA levels in patients with ulcerative colitis were found to correlate with the severity of the disease (58). An enhanced expression of ADA was also observed in animal models of experimental colitis (59, 60). Of note, treatment with ADA inhibitor alleviated the severity of inflammation in animals with colitis (60–63). These findings suggest that ADA could represent a potential diagnostic marker as well as therapeutic targets in the treatment of IBDs. Indeed, the simplicity to evaluate the ADA expression and activity associated with a good cost effectiveness ratio represent elements in favor of using this enzyme as a useful inflammatory biomarker in IBD patients, despite this additional controlled studies are needed to further corroborate the role of ADA as an independent index of inflammation in IBDs.

As previously described, the nucleoside transporters actively participate in maintaining the adenosine levels in the extracellular space. In this regard, Wojtal et al. observed that colonic tissues obtained from patients with IBDs displayed increased mRNA levels of ENT1, ENT2, and CNT2 mRNA, thus leading to hypothesize a reduced bioavailability of endogenous adenosine (64). Interestingly, Aherne et al. reported that the administration of dipyridamole, a ENT 1 and ENT2 blocker, exerted protective effects in a murine DSS model of colitis (65). In this context, the ENT1 gene deletion did not counteract the progression of colitis, while ENT2 gene deletion was protective against intestinal inflammation, suggesting a critical involvement of ENT2 in the onset and development of bowel inflammation (65). The mechanisms underlying the anti-inflammatory effects of ENT2 inhibition or deficiency resulted from the increased levels of extracellular adenosine that exerted its protective effects through A_2B_ receptor activation (65). Unfortunately, no data are available about the beneficial effects of a pharmacological modulation of ENT2 in other murine models of colitis, not allowing a comprehensive evaluation of its efficacy in intestinal inflammation supported by other immune paradigms.

Overall, current human and pre-clinical evidence support the contention that pharmacological modulation of purinergic pathways is a suitable therapeutic approach for the treatment of bowel inflammation. In particular, A_2A_ and A_3_ receptor agonists displayed beneficial effects in intestinal dysfunctions associated with inflammatory bowel disorders, including visceral pain, diarrhea, ischemia and functional disorders. However, the role of purinergic system in the modulation of digestive functions still remains poorly understood and deserves extensive future investigations.

### Role of Adenosine System in Abdominal Pain

Abdominal pain is a symptom frequently associated with the presence of IBDs (66). Indeed, a number of patients in the acute phase of IBD will experience pain, typically improving upon disease activity decrease (66). Of note, a large part of IBD patients continue experiencing pain also under clinical remission (66).

Over the past years, huge efforts have been addressed to characterize the role of the endogenous mediators released during enteric dysfunctions and involved in pain perception (1). In this regard, adenosine receptors are actively involved in the rearrangement of enteric sensory pathways (1).

At present, the role of adenosine in the pathophysiology of visceral pain has been scarcely deepened and often the available evidences are conflicting (67, 68). Pre-clinical studies pointed out an inhibitory effect exerted by adenosine, via A_1_ receptor activation, on pain transmission both at pre-synaptic level, counteracting the pain-associated neurotransmitter release, such as glutamate, calcitonin gene-related peptide and substance P, and at post-synaptic level, through membrane cell hyperpolarization (68, 69). Sohn et al. reported that the intrathecal administration of the A_1_ receptor agonist R-PIA, but not the A_2A_ receptor agonist CGS-21680 hydrochloride, decreased the visceromotor responses (70). Currently, some authors paid greater attention to the potential anti-nociceptive effects of A_3_ agonists (71, 72). For instance, Hou et al. demonstrated the analgesic effects of A_3_ receptor agonists in a mouse model of visceral pain following experimental colitis (71). At present, no data are available about the putative analgesic effect of A_2A_ ligands on abdominal pain associated with experimental colitis. This is an intriguing point to address, since as previously described, the A_2A_ agonists are actively under evaluation for IBD management based on their marked immunomodulatory effects.

### Role of Adenosine System in Enteric Dysmotility Associated With IBD

Over the years, increasing efforts have been addressed to unravel the link between the enteric inflammation and the neuronal alterations in the digestive tract. Inflammation-induced changes occur in several neuronal compartments, including the sympathetic prevertebral ganglia, the dorsal root ganglia, and the enteric ganglia (73).

In this context, the evaluation about the involvement of adenosine pathways in the pathophysiology of enteric dysmotility associated with IBDs, has become an area of active investigation (1, 74, 75). Several evidences highlighted a marked reorganization of adenosine receptor expression and activity in the presence of intestinal inflammation (1, 17, 74, 75). Different murine model of chronic bowel inflammation revealed a reduced modulatory role by A_1_ receptors in the small and large bowel (76, 77). The loss of A_1_ receptor activity has been ascribed to a sustained exposure at marked concentrations of adenosine, leading to a receptor desensitization (14). A reduced inhibitory modulation via A_1_ receptors on colonic cholinergic responses have been observed in a rat model of DNBS-colitis. However, the authors related this event to an increased degradation of endogenous adenosine, with a consequent reduction of its bioavailability, rather than to a receptor desensitization (14). Accordingly, Antonioli et al. reported a limited A_1_ receptor activation arising from a site-specific production of adenosine, operated by the enzyme CD73, preferentially in the A_2A_ receptor biophase (14), previously reported as critically involved in the modulation of colonic nitrergic transmission in DNBS-treated rats (78). In the presence of intestinal inflammation, a reorganization of the receptor expression and function as well as the presence of functional interplays with metabolic pathways, have been described also for A_2B_ receptors (59). Indeed, the inhibitory control exerted by A_2B_ receptors on colonic contractile responses was impaired in the presence of experimental colitis, despite an up regulation of such receptors in the colonic neuromuscular layer from inflamed animals (59). Molecular investigations demonstrated the co-localization of adenosine deaminase with the A_2B_ receptor, suggesting a functional interplay, where adenosine deaminase, catabolizing the endogenous adenosine, reduced A_2B_ receptor activation (59). In accordance, adenosine deaminase has also been shown to play a modulatory role in the activity of the A_3_ receptor in the inflamed colon (79).

Analogously to what was reported for A_2B_ receptors, the presence of colonic inflammation was characterized by the loss of the A_3_ receptor inhibitory activity, an up-regulation of functioning A_3_ receptors occurred (79). This altered A_3_ receptor expression occurred concomitantly with an increase in adenosine deaminase expression in the colonic neuromuscular compartment of rats with colitis, thus decreasing the bioavailability of endogenous adenosine in the A_3_ receptor microenvironment (79). Based on these evidences, the pharmacological blockade of adenosine deaminase may represent an intriguing strategy to limit the inflammatory process and contextually counteract the enteric motor alterations typically observed in IBD patients.

## Concluding Remarks

The etiopathogenesis of IBD is still poorly understood, despite a number of recent evidences revealing that enhanced knowledge about the immunological mechanisms underlying IBD onset and progression represent an interesting target to design and synthesize innovative therapeutic strategies (80). The current available pharmacological options are effective, but unfortunately some of these drugs displayed marked adverse events, such as infections or an enhanced risk of neoplastic diseases or they lose their effectiveness over time. Indeed, about one third of the patients show a slight response to these therapies (80).

A number of pre-clinical studies revealed the involvement of the adenosine system in the modulation of immune, functional and sensory systems of the gastrointestinal tract (81). In this regard, an increasing interest has been focused toward the A_2A_ and A_3_ receptor agonists as interesting targets to generate novel pharmacological entities useful to manage the digestive dysfunctions. Indeed, the use of selective A_2A_ or A_3_ receptor agonists showed beneficial effects in counteracting the inflammatory burst in murine models of colitis, acting on both the innate and acquired component of the immune system (82–85). In parallel, the stimulation of such receptor subtype revealed to exert a significant role in the regulation of colonic neuromuscular activity in the presence of bowel inflammation (77, 79). In particular, the engagement of A_2A_ or A_3_ receptors by selective agonists appear to be an interesting method of management for IBD patients displaying an increased gut motility and diarrhea (77, 79). A number of encouraging data are emerging about the modulatory role of adenosine receptors on visceral sensitivity (71, 72). The A_3_ receptor agonists highlighted a pain-relieving mediated through N-type Ca^2+^ channel block and action potential inhibition, suggesting the A_3_ receptor agonists as an innovative approach to manage the visceral pain (59).

These data spurred the interest of the scientific community toward the development of novel ligands acting selectively on adenosinergic receptors/enzymes. These novel pharmacological tools will allow to better deepen the pathophysiological meaning as well as the putative therapeutic relevance of the adenosine pathway, paving the way to the development of novel therapeutic options useful for the treatment of IBDs.

## Author Contributions

LA, MF, CP, LB, ZN, and CB participate to bibliographic research, to write and revised the manuscript. All authors contributed to the article and approved the submitted version.

## Conflict of Interest

The authors declare that the research was conducted in the absence of any commercial or financial relationships that could be construed as a potential conflict of interest. The handling editor declared a past co-authorship with one of the authors LA.
